# Crouching Tiger, Hidden Trouble: Urban Sources of *Aedes albopictus* (Diptera: Culicidae) Refractory to Source-Reduction

**DOI:** 10.1371/journal.pone.0077999

**Published:** 2013-10-22

**Authors:** Isik Unlu, Ary Farajollahi, Daniel Strickman, Dina M. Fonseca

**Affiliations:** 1 Center for Vector Biology, Rutgers University, New Brunswick, New Jersey, United States of America; 2 Mercer County Mosquito Control, West Trenton, New Jersey, United States of America; 3 USDA Agricultural Research Service, Office of National Programs, Beltsville, Maryland, United States of America; Kansas State University, United States of America

## Abstract

Our ultimate objective is to design cost-effective control strategies for *Aedes albopictus*, the Asian tiger mosquito, an important urban nuisance and disease vector that expanded worldwide during the last 40 years.  We conducted mosquito larval surveys from May through October 2009 in the City of Trenton, New Jersey, USA, while performing intensive monthly source-reduction campaigns that involved removing, emptying, or treating all accessible containers with larvicides and pupicides. We examined patterns of occurrence of *Ae. albopictus* and *Culex pipiens*, another urban mosquito, among different container types by comparing observed and expected number of positive containers of each type. Expected use was based on the relative frequency of each container type in the environment. *Aedes albopictus* larvae and pupae were found significantly more often than expected in medium volumes of water in buckets and plant saucers but were rarely collected in small volumes of water found in trash items such as discarded cups and cans. They were also absent from large volumes of water such as in abandoned swimming pools and catch basins, although we consistently collected *Cx. pipiens* from those habitats. The frequency of *Ae. albopictus* in tires indicated rapid and extensive use of these ubiquitous urban containers. Standard larval-based indices did not correlate with adult catches in BG-Sentinel traps, but when based only on *Ae. albopictus* key containers (buckets, plant saucers, equipment with pockets of water, and tires) they did. Although we found that only 1.2% of the 20,039 water-holding containers examined contained immature *Ae. albopictus* (5.3% if only key containers were counted), adult populations were still above nuisance action thresholds six times during the 2009 mosquito season. We conclude that in urban New Jersey, effective source reduction for *Ae. albopictus* control will require scrupulous and repeated cleaning or treatment of everyday use containers and extensive homeowner collaboration.

## Introduction

Mosquitoes that oviposit and develop in containers, such as several invasive *Aedes* and *Culex* species, are commonly the primary nuisances and pathogen vectors in large urban areas [1,2]. The heterogeneity of containers, which ranges from tree holes to backyard pools to drinking cups, tires, and catch basins, poses a major challenge for mosquito abatement programs. The current paradigm is that unlike wetland mosquito species that oviposit and develop in habitats that are large, predictable, and easy to identify, the numerous small habitats used by container-inhabiting species are difficult to locate and control [3]. This problem has led to intense research into the determination of the most productive and preferred containers by these mosquitoes in order to direct effective control strategies [4-7]. Studies from several urban areas indicate that *Aedes aegypti* (L.) prefers containers holding drinking water, especially those retained near or inside homes [4,8-10], while *Culex pipiens* L. and *Cx. quinquefasciatus* Say prefer larger water bodies with higher organic matter and are often found in abandoned pools, catch basins, and sewers [11-13]. In contrast, *Aedes albopictus* (Skuse) is often found in a remarkably diverse array of containers [7,14,15] including trash items such as small cups and cans, implying the need for exhaustive source reduction a labor intensive and costly strategy.

A better understanding of preferred or highly productive containers should allow the use of shortcuts such as entomological indices based on larval abundance, to pinpoint heavy infestations timely and prioritize the use of limited resources [16]. Three indices that have been extensively used in programs to control *Ae. aegypti* are the House Index (HI), defined as the percentage of houses that are positive for larvae, the Container Index (CI), defined as the percentage of water-holding containers that are positive for larvae, and the Breteau Index (BI), defined as the number of mosquito positive containers per 100 houses. Although the predictive power of these indices regarding adult populations of *Ae. aegypti* and disease transmission is still highly debated, as summarized by Focks & Chadee [4], Thammapalo et al. [17], and Sanchez et al. [18], their use has recently been expanded for *Ae. albopictus* surveillance [19,20]. 

Because of its relatively recent worldwide expansion and emergence as an important disease vector [21,22] less is known about the larval habitat preferences of *Ae. albopictus* than those of *Ae. aegypti* or *Cx*. *pipiens/quinquefasciatus*, which have been important vectors of deadly and debilitating diseases to humans for a long time. Published larval and pupal surveys of *Ae. albopictus* in Japan, Thailand, La Réunion, Cameroon, Italy and USA [5-7,14,23-27] report the species in a wide variety of containers including coconut shells, dead cow horns, abandoned cars, tires, plant saucers, and even bottle caps. Wheeler et al. [15] associated their failure to control *Ae. albopictus* with techniques that had been successfully used to eradicate *Ae. aegypti*, to differences in oviposition preferences between the two species. Estrada-Franco and Craig [28] stated that it is more difficult to control or eliminate *Ae*. *albopictus* than *Ae. aegypti* because *Ae*. *albopictus* is found farther from human habitation and in a wider range of habitats. 

Indeed, it has been proposed that the global expansion of this species may be related to its successful adaptation to a variety of artificial containers [29,30]. Since its initial introduction to the United States in the 1980’s, the species has spread throughout the southeastern and eastern USA [3,22,29,31]. In New Jersey, *Ae. albopictus* was first detected in 1995 [32] but recently its populations have increased dramatically within the very urbanized northeastern USA and even though it has not become an important local disease vector yet, it is a major nuisance impacting Public Health [3,22,33,34]. 

Because of the vast array of potentially important containers, *Ae. albopictus* larval surveillance and control is thought to require labor-intensive, continual, and costly source reduction of all artificial containers in urban habitats [3,33,35]. However, even if any container may, at times, contain immature *Ae. albopictus*, it is possible that some container types are more important sources than others especially if they are refractory to source-reduction. If that is the case, directing control efforts to the sub-set of container types more likely to be sources of adults should lead to more cost-effective control strategies. Our objectives were to (1) assess overall and temporal patterns of occurrence of *Ae. albopictus* in different container types during all-season intensive source reduction campaigns in a low socio-economic urban setting with an abundance of water-holding containers; (2) identify container types refractory to treatment (i.e. consistently positive for *Ae. albopictus*); (3) examine the effect of container type on the correlation between larval indices and the number of adult *Ae. albopictus* collected by a local array of BG-Sentinel traps™ (BGS) traps. We performed quasi-exhaustive surveys of water-holding containers in an urban setting during monthly intensive source reduction campaigns that involved removal, emptying, or application of larvicides and pupicides to all accessible containers. To provide a measure of the power of our analyses we also examined occurrence patterns of immature *Cx. pipiens* in our sampling sites. Our ultimate objective is to design cost-effective control strategies against *Ae. albopictus*.

## Materials and Methods

### Ethics statement

No specific permits were required for the described field studies, which were developed with homeowners assent by professional county mosquito control personnel. These studies did not involve endangered or protected species.

### Study sites, container treatment, and sampling methodology

**Figure 1:**
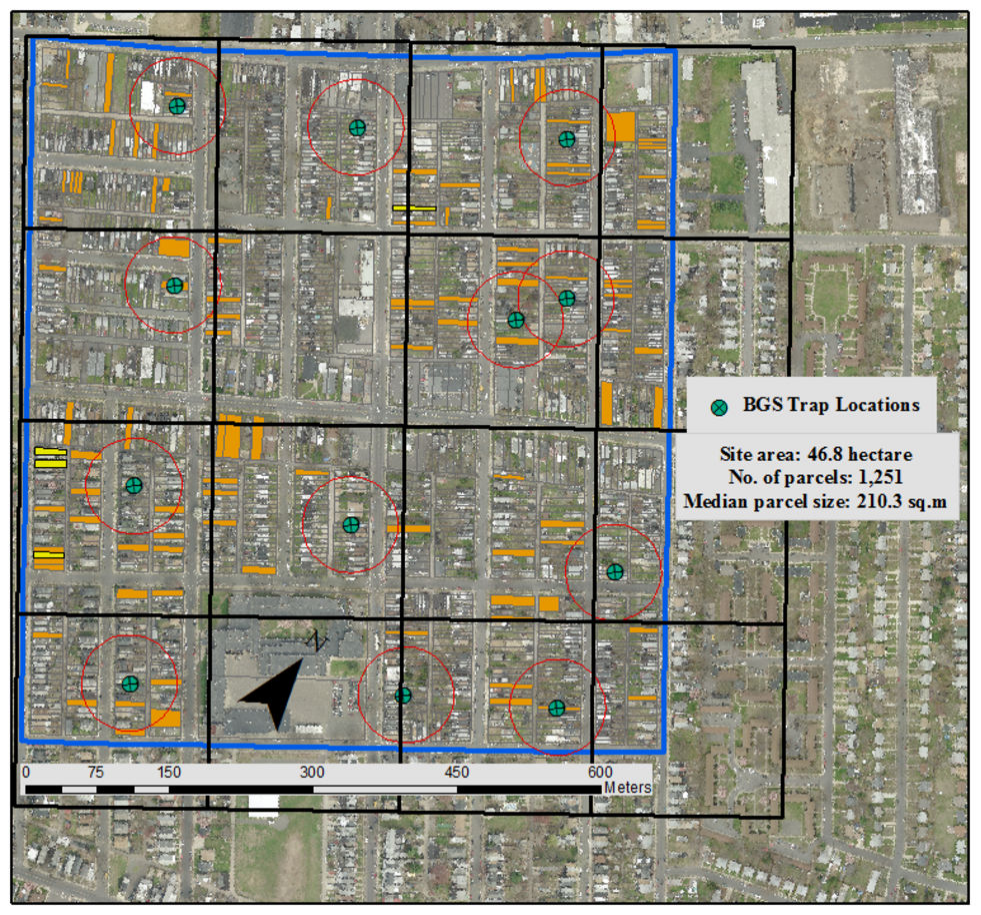
Aerial map of the study site and 12 BGS trapping sites within a 50 m radius (red circles/sampling units). We aimed to place each BGS trap within one of the cells of a square grid with 200 m sides. When that was not possible we placed the BGS trap in a nearby cell. Orange highlighted parcels indicate parcels with at least one container positive for immature *Ae. albopictus* during the month of August 2009. The four parcels highlighted in yellow were the only ones never inspected during the study.

Treatment of containers and surveys of immature mosquitoes were conducted between May and October 2009 in the City of Trenton, Mercer County, New Jersey, USA. The study area was 48.6 hectares (centered on 40° 22’N, 74° 73’W) in a densely urban neighborhood with 1,250 parcels (i.e., house and corresponding yard) divided into 24 city blocks of row homes, businesses, and a school. The site was divided into 78 sampling zones based on subdivisions of city blocks (Figure 1). Parcel sizes were relatively constant at approximately 200 m^2^ [35,36]. The area was characterized by few large trees and many vacant or neglected properties (6.5% of all parcels) that often had yards with over-grown vegetation. Surveillance and treatment reoccurred monthly on a series of consecutive days (weather permitting and weekends excepted) until the entire area had been surveyed and treated. Treatment involved source reduction (SR, i.e. removal or emptying of containers) and hand-application of a combination of larvicide and pupicide [37] to the remaining containers. This study was part of a USDA sponsored project for area-wide management of the Asian tiger mosquito (USDA-ARS-58-6615-8-105). For extensive details regarding the source-reduction and treatment strategies employed please refer to Fonseca et al. [35] where there is also a comparison between adult populations of *Ae. albopictus* in the site that was the focus of the present study (full intervention site) and a paired untreated control site (no intervention). Survey and treatment of all containers in accessible private parcels required 14±5.1 days/month using two to five teams of three trained personnel each. All properties within the site (i.e. residential, abandoned, and commercial) as well as alleyways were included, except for parcels whose owners refused access or that were inaccessible because of physical barriers (fallen structure). We initiated work with a brief explanation to the residents of the purpose of the study, inspected both front and backyards in search of larvae-positive containers, performed source reduction when possible, and finally implemented the needed treatments. In addition, and as part of routine Mercer County control of *Cx. pipiens*, an important urban arbovirus vector of West Nile virus in the northeastern US [38], all catch basins were treated with a combination of VectoBac®12AS and VectoLex® WDG (Valent BioSciences, Libertyville, IL, USA) twice a month from early July until the end of September 2009.

### Data collection and species identification

**Figure 2:**
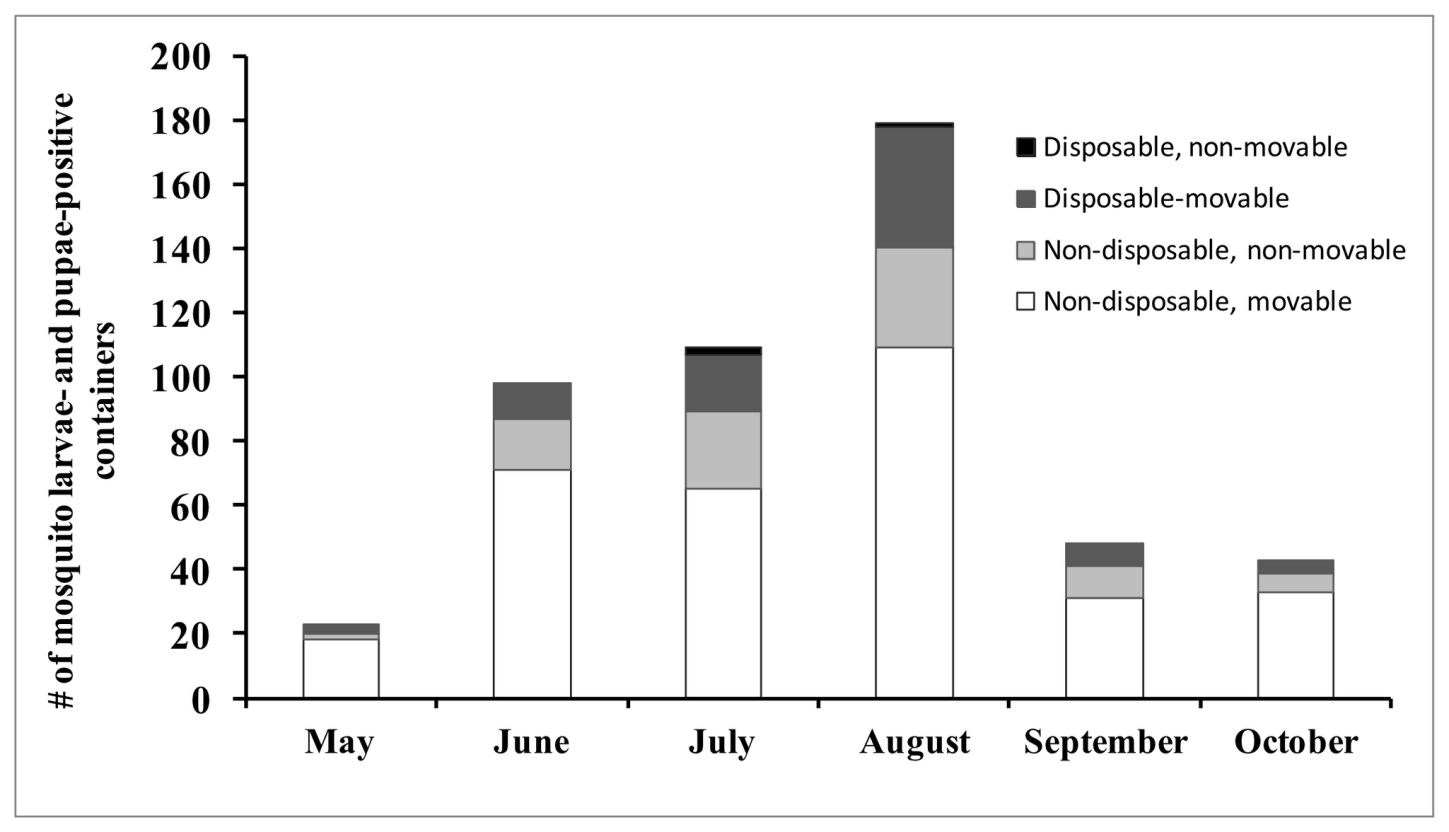
Number of wet containers and container type examined each month (May-October) in the study site during larval surveys in 2009. Containers were classified as “disposable” if intended for one-time use, “non-disposable” if intended for long-time term or repeated use, “movable” if easily displaced by an average adult and “non-movable” if otherwise.

Containers holding water were examined for the presence of mosquito larvae and pupae. We used a mosquito dipper for deep containers, such as recycle bins, and a suction device (“turkey baster,” a 20 cm long x 5 cm wide plastic tube with a narrow opening at one end and a suction bulb at the other) with a 0.25 L capacity for slender containers, such as hollow fence posts and narrow tires. We poured water from containers into a white plastic tray to increase visibility of larvae and pupae. When a container held large numbers of larvae, a sub-sample (at least 10 larvae or pupae) was taken and placed in a 500 ml capped vial. We recorded several environmental factors associated with each container. First, we estimated the volume of water in four categories that were easy to use by field personnel: (1) small: containers containing 40 -250 ml of water, (2) medium: containers containing >250 ml -1L, (3) large: containers containing, >1 - 20L, and (4) very large: containers containing > 20L. These volumes were the estimated amount of water in the containers; therefore a 4L bucket could be classified under the first category (40-250 ml) for water volume classification. We provided simple guidelines to the field crews to help classify water volume more easily, such as a volume between a drinking cup and soda can in order to identify a small volume of water in containers, etc. Following the guidelines of Bartlett-Healy et al. [39] we classified containers as (a) “disposable” if intended for one-time use, such as paper plates, snack bags, or cans; (b) “non-disposable” if intended for long-term and repeated use, such as buckets, or gutter extensions; (c) “conceivably movable” such as an empty garbage container, toys, or bowls; (d) “non-movable “if the object was fixed or heavy, such as gutters, bird baths, automobiles, and discarded toilets (Figure 2). In addition, we recorded amount of exposure (full sun, partial shade, and full shade), and organic content of water (high, medium, low). Exposure was determined as: (a) “full sun” if with direct sunlight all day, (b) “partial sun” if with direct sunlight during half of the day, and (c) “shade” if with no direct sunlight. Organic matter was scored as “high” (>10 leaves and other organic matter in the container), “medium” (4-9 leaves and some other organic matter), or “low” (< 3 leaves and no other visible organic matter). The color of the water and the presence of debris were used to score organic matter in the containers. We labeled each larval and pupal sample and data sheet with a unique number for later identification. Larval species and developmental stages were identified using established keys [2,40]. Our main objective was to develop a near-exhaustive survey of the occurrence of immature mosquitoes in various types of containers and assess the usefulness of standard indices that rely on presence-absence counts, therefore we did not attempt to quantify or estimate mosquito abundance. 

### Larval and Pupae Indices and adult abundance

We calculated the match between larvae and pupae based indices and adult abundance, from an array of 12 BGS traps (Biogents AG, Regensburg, Germany) deployed as part of a USDA-funded “Area-wide Management of the Asian tiger Mosquito” project [35,38,41,42]. The BGS trap was chosen as our adult surveillance tool because of its proven efficiency for capturing adult *Ae. albopictus* [43]. We calculated the larval and pupal indices (HI, CI, and BI) for parcels within a 50m radius from each BGS trap location (Figure 1, 12 BGS trapping locations with an average of 36 parcels ± 2/sampling unit) during the month of August, the peak time for both number of *Ae. albopictus* larval- and pupal-positive containers as well as adult populations [35]. Shape files for parcels and the study site were projected using the North American Datum 1983 coordinate system [44,45] to determine the boundaries of sampling units and 50 meter radius sampling units around each trapping location was created using the “buffer wizard tool” within ARCMap (ESRI^™^, Redlands, CA, USA). The study site is 600 m x 600 m, and therefore a 50 m radius around each BGS trap was chosen in order to maximize the number of parcels included in the analysis without overlap and without including parcels outside the study site. We also calculated these indices by restricting the container types to those we found to have significant positive association with presence of *Ae. albopictus* larvae and pupae; bucket, plant saucer, equipment and tire (see Results). We designate these as “key” containers henceforth.

### Meteorological data

**Figure 3:**
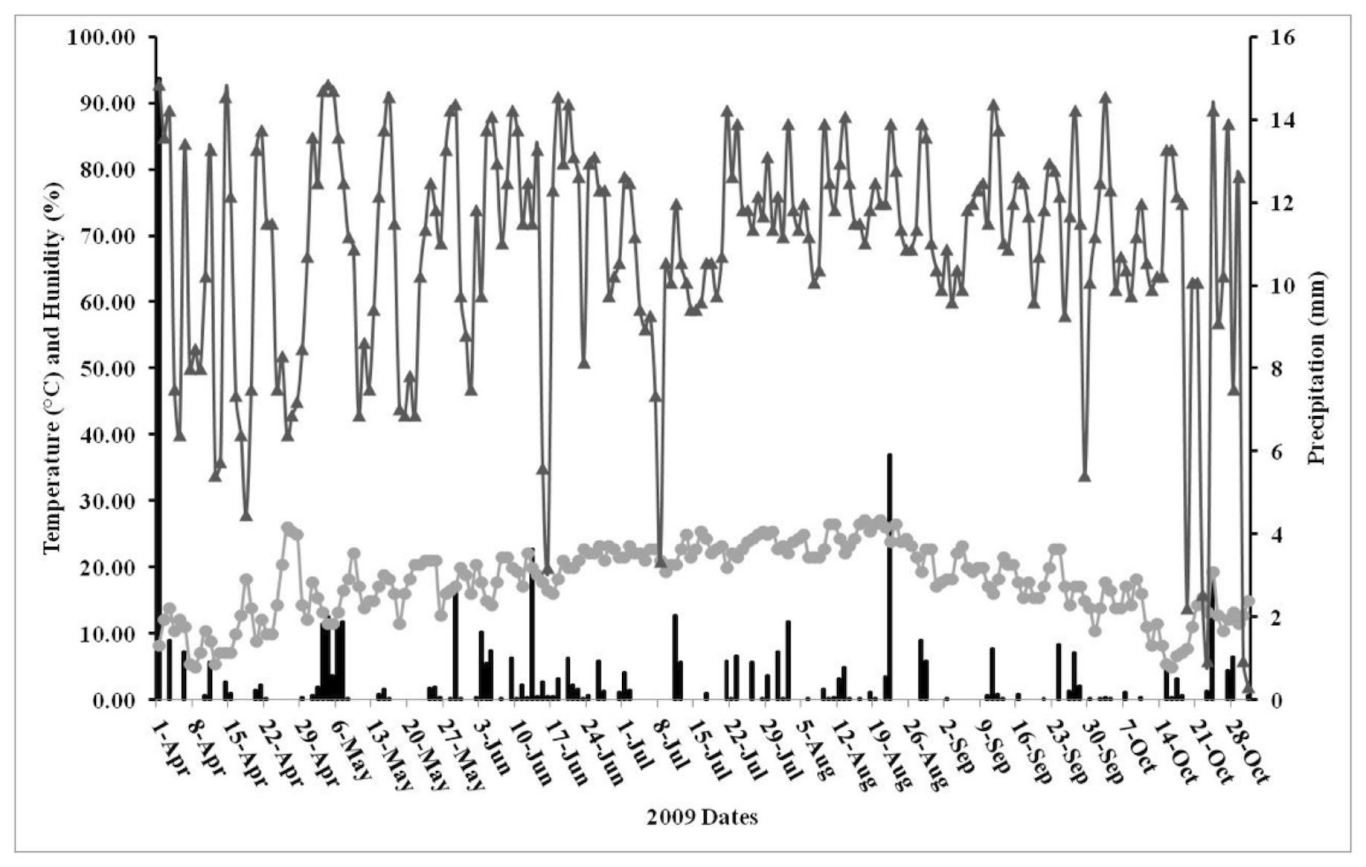
Daily temperature, humidity, and rainfall data from the Trenton weather station (Mercer-Trenton Airport, TTN) during the 2009 mosquito season. Gray line with circles: temperature, dark grey line with arrows: humidity, black columns: precipitation.

Daily temperature, humidity and rainfall data were obtained from the New Jersey Weather Climate and Network and Trenton weather station (Office of the New Jersey State Climatologist, Department of Geography, Rutgers University, ONJSC) (Figure 3). Additional meteorological data was obtained from a permanent weather station located at Trenton-Mercer Airport, situated 7.5 km from the surveillance site.

### Data analysis

We classified more than 100 different types of containers into 13 major categories based on their size and characteristics: bowl, bucket, catch basin, equipment, gutter, gutter extension, natural container, plant saucer, pool, small trash (any item that was considered as recyclable or not re-used by home owners such as bottles, bottle caps, cups, cans, plastic bags, snack bags, tarp, tire, and small toy; please refer to the legend of Table 1 for more details) and examined both the seasonal pattern of occurrence of different container types and the occurrence of immature *Ae. albopictus* and *Cx. pipiens*, the two most common mosquito species. Because both container type and presence and absence of larvae are qualitative variables we assessed the significance of a positive or negative association by comparing the observed and expected number of positive containers of each type for each species using χ^2^ analyses. The expected number of positive containers of each type was based on the frequency of occurrence of each container type. A lack of significant association indicated that the mosquito species occurred in a container type at a frequency that matched the occurrence of that container in the environment. A significant association indicated that the species was collected in a container type more (or less) often than expected from the frequency of occurrence of that container type in the environment. A significant positive association indicated preference (rapid colonization of new containers of that type) or higher survivorship (due to failure of control) and a significant negative association indicated avoidance or lower survivorship in specific types of containers.

**Table 1:** Summary of the occurrence of *Aedes albopictus* in the different container types.

Containers^a^	No. with *Ae. albopictus* /No. inspected (%)	No. with *Ae. albopictus* and another species^b^ /No. inspected (%)
Bowl	18/570 (3.2)	6/570 (1.1)
Bucket	87/1460 (6.0)	35/1460 (2.4)
Catch basin	3/34 (8.8)	0/34 (0.0)
Equipment	16/312 (5.1)	5/312 (1.6)
Gutter	1/229 (0.4)	1/229 (0.4)
Gutter extension	6/333 (1.8)	1/333 (0.3)
Natural Container	1/15 (6.7)	0/15 (0.0)
Plant saucer	23/472 (4.9)	6/472 (1.3)
Pool	2/129 (1.6)	0/129 (0.0)
Small trash	31/9,327 (0.3)	4/9,327 (0.0)
Tarp	11/554 (2.0)	3/554 (0.5)
Tire	22/554 (4.1)	6/544 (1.1)
Toy	10/475 (2.1)	1/475 (0.2)
Other^c^	9/5,583 (0.2)	2/5,583 (0.0)
**Total**	**241/20,039 (1.2)**	**70/20,039 (0.3)**

a Over 100 different types of containers were inspected and summarized into the 13 categories listed above. The container type often reflects the name of the container. But seven of the categories include containers that provide comparable larval habitats as follows: “Bowl” includes pots, pans, animal bowls; “Bucket“ includes bins, recycle bins, trash cans; “Equipment” includes appliances, wheel barrows, cement mixers; “Natural container” includes tree holes, stumps, puddles; “Pool” includes abandoned cement pools, large plastic pools and ornamental ponds; “Small trash” includes bottles, cups, cans, plastic bags, snack bags; and “Toy” includes plastic cars, kiddie pools.

b Co-existence of *Ae. albopictus* with one or more of these following species: *Ae. japonicus, Ae. triseriatus, Cx. pipiens, Cx. restuans, Ps. ferox, Tx. rutilis septentrionalis*.

c Inspected containers were categorized as “other” when field notes did not indicate a specific container type.

The association between presence of larvae and pupae of each species was assessed using a nominal logistic regression and the significance values were based on maximum likelihood Wald test statistics (equivalent to a χ ^2^ – test) [46]. We also tested the overall significance of the association between presence and absence of *Ae. albopictus* in each container type with a maximum likelihood Wald test but could not do the same for *Cx. pipiens* because of their low relative occurrence. We calculated the correlation between larval indices calculated within each 50m radius around a BGS trap and the number of adult *Ae. albopictus* caught in the BGS trap using least-squares linear regression. All statistical analyses were performed with JMP® (SAS Institute Inc, 2008, Cary, NC, USA).

We created four temporal stages adjusted to allow for the low number of positive containers early and late in the season. The stages were: Stage 1: May-June; Stage 2: July; Stage 3: August; and Stage 4: September-October. We examined the correlation between the occurrence of *Ae. albopictus* and that of *Cx. pipiens* across all four time stages. 

## Results

We were able to gain access, inspect, and treat 1,248 of the 1,251 parcels in the study site (Figure 1). 66.8% of the parcels were inspected and treated during all five monthly source reduction actions. 83.3% were inspected four times, 94.6% three times, 98.5% twice, and 99.7% at least once. Overall, our actions resulted in a decline in the abundance of adult *Ae. albopictus* in the treated site by more than 75% compared to a matched untreated site [35], indicating a significant impact of the treatments we implemented. A wide variety of container types were present in the community (Table 1). The most abundant containers were small trash items (46.5%) and the least abundant were tree holes (0.1%), which were the only natural containers. The second most abundant containers we encountered were plastic buckets (7.2%), followed by bowls (2.8%), tarps (2.7%), and tires (2.8%). We found the greatest number of water-holding containers during the early season and late season surveys (Table 2). Of note, August 2009 was the 6^th^ warmest month on record for New Jersey and the summer of 2009 was the 5^th^ wettest summer on record based on observations in Trenton, NJ, since 1895 (Office of the New Jersey State Climatologist) and as a result, containers with some water (“wet containers”) were very common throughout the season. 

**Table 2:** Seasonal patterns of occurrence of containers defined as “key” for *Ae. albopictus* (the first 4 types) as well as a summary of abundance of other containers.

Container type	Apr	% A	May	% A	Jun	% A	Jul	% A	Aug	% A	Sept	% A	Oct	% A
Bucket	230	0	79	2.5	239	2.4	351	4.3	147	31.9	154	5.2	260	5.3
Plant saucer	15	0	49	0	62	0	94	1.1	148	9.5	36	16.6	68	2.9
Tire	20	0	147	1.5	97	0.9	85	3.5	115	13.9	23	0	57	0.6
Equipment	1	0	64	0	38	0	56	5.3	85	9.4	33	15.2	35	0
Other	1872	0	5904	0	2183	0.2	1721	0.8	1314	3.5	1164	1.2	3093	0
**Total**	**2138**		**6243**		**2619**		**2307**		**1809**		**1410**		**3513**	

Also shown are percent containers of each type positive for *Ae. albopictus* (% A). For details regarding the assignment of a container to a “type” please refer to Table 1 and the text.

Of 20,039 wet containers inspected, 569 (2.8%) were found positive for mosquito larvae. In all, we identified eight mosquito species, but *Ae. albopictus* was the most common and was collected in 241 (42.3%) of the mosquito-positive containers (Table 1). *Culex pipiens* was the second most common species, occurring in 151 containers (26.5%), followed by *Culex restuans* Theobald in 131 containers (23%), *Aedes* j. *japonicus* (Theobald) in 106 containers (18.6%), and *Aedes triseriatus* (Say) in 16 containers (2.8%). Six containers were positive for *Psorophora ferox* (von Humboldt), *Toxorhynchites rutilus septentrionalis* (Dyar and Knab), *Aedes atropalpus* (Coquillett), and unidentified *Culex* species. *Aedes albopictus* was discovered co-existing with *Ae*. *j. japonicus* in 7.6% of the positive containers and with either *Cx. pipiens* or *Cx. restuans* in 6.7% of the positive containers (Table 3). Ninety percent of the mosquito-positive containers surveyed in this study contained *Ae. albopictus, Cx. pipiens, Cx. restuans*, or a combination of at least two of these three species (Table 2 and 3). *Aedes* j. *japonicus* larvae were more prevalent than *Ae. albopictus* in May and June but then decreased in presence (data not shown). There was only one collection of *Ae. atropalpus* during this survey, in a tire exposed to direct sunlight. Of fifteen tree holes surveyed, only one contained mosquito larvae, and they were *Ae. albopictus*. 

**Table 3:** Occurrence of other mosquito species that exploit containers during immature stages in urban northeastern US.

Containers	No. with *Cx*. *pipiens*/No. inspected (percentage)	No. with *Cx*. *restuans*/No. inspected (percentage)	No. with *Ae*.. *japonicus*/No. inspected (percentage)
Bowl	10/570 (1.8)	5/570 (0.9)	11/570 (1.9)
Bucket	61/1,460 (4.2)	81/1,460 (5.25)	47/1,460 (3.2)
Catch basin	14/34 (41.2)	12/34 (35.3)	0/34 (0)
Equipment	9/312 (2.9)	3/312 (1)	8/312 (2.6)
Gutter	0/229 (0)	0/229 (0)	1/229 (0.4)
Gutter extension	1/333 (0.3)	0/333 (0)	1/333 (0.3)
Natural container	0/15 (0)	0/15 (0)	0/15 (0)
Plant saucer	6/472 (1.3)	3/472 (0.6)	15/472 (3.2)
Pool	16/129 (12.4)	6/129 (4.7)	1/129 (0.4)
Small trash	6/9,327 (0.1)	2/9,327 (0)	5/9,327 (0.8)
Tarp	9/554 (1.6)	7/554 (1.3)	5/554 (1)
Tire	12/566 (2.1)	12/566 (2.1)	7/566 (1.2)
Toy	6/4,75 (1.3)	0/475 (0)	3/475 (0.6)
Other	1/5,563 (0)	0/5,563 (0)	2/5,563 (0)
**Total**	**151/20,039 (0.8)**	**131/20,039 (0.7)**	**106/20,039 (0.5)**

Immature *Ae*. *albopictus* were most often collected in buckets (87 buckets were positive in all of the surveys) within the study area, though only 6% of buckets holding water contained larvae or pupae (Table 1). A low frequency of occurrence of mosquito larvae was a common pattern throughout the surveys (Table 1, Table 2). During the early spring survey (before source-reduction and treatments started) *Ae. albopictus* larvae were only collected from tires and buckets (100%, Table 2), whereas *Cx. pipiens* were primarily collected from buckets, catch basins, and abandoned pools (72.4%, Table 3). Interestingly, despite our intervention this general pattern did not change markedly (Table 2). *Aedes albopictus* displayed a non-random distribution across wet containers (Wald χ^2^ =222.4; df =12; *P* =0.01) (Table 4) being present more often than expected by chance (based on frequency) in buckets (χ^2^ =25.5; df =12; *P* <0.01), plant saucers (χ^2^ =7.8; df =12; *P* <0.05), and small water pockets in equipment (χ^2^ =7.1; df =12; *P* < 0.05). Although we identified 25 abandoned pools and 2 ornamental ponds that were positive for mosquito larvae, *Ae. albopictus* were never collected from these habitats. Our study also revealed that *Ae*. *albopictus* was rarely found in small trash (χ^2^ = 85.6; df =12; *P* <0.01), and roof gutters (χ^2^ = 3.6; df =12; *P* <0.05) in our sampling site. 

**Table 4:** Detailed presence data for *Ae*. *albopictus* and *Cx. pipiens*.

Container type	with (without) *Ae.albopictus*	expected^a^ *Ae. albopictus*	with (without) *Cx. pipiens*	expected^a^ *Cx. pipiens*	Total#^c^
Bowl	18 (552)	9	10 (560)	5.9	570
Bucket	87* ^b^ (1,373)	23	61* (1,399)	15.1	1,460
Catch basin	3* (31)	0.5	14* (20)	0.3	34
Equipment	16* (296)	4.9	9 (303)	3.2	312
Gutter	1** ^b^ (228)	3.6	0** (229)	2.3	229
Gutter extension	5 (328)	5.2	1 (332)	3.4	333
Natural container	1 (14)	0.2	0 (15)	0.2	15
Plant saucer	23* (449)	7.4	6 (466)	4.8	472
Pool	2 (127)	2	16* (113)	1.3	129
Small trash	31** (9,296)	147	6** (9,321)	96.6	9,327
Tarp	11 (543)	8.7	9 (545)	5.7	554
Tire	22 (544)	8.9	12 (554)	5.8	566
Toy	9 (466)	7.5	6 (469)	4.9	475

a Expected number of *Ae*. *albopictus* and *Cx. pipiens* larvae/pupae positive containers based on contingency analysis taking into consideration frequency of container type among all containers with water inspected.

b Significant deviations from expected are marked with an asterisk (one if found more often than expected and two if less often than expected).

c Total number of containers of each type inspected during the surveys.

The presence of *Ae*. *albopictus* was non-random with regards to water volume (χ^2^= 19.9; df=3; *P* < 0.05) (45.2%) since the species was found significantly more often than expected in medium sized volumes of water (250 ml - 1L, χ^2^= 7.8; df=3; *P* < 0.05). Although the majority of containers positive for *Ae. albopictus* larvae and pupae were located in the shade or partial shade (63.2%) with only 26.2% in full sun, this pattern was not significantly different from random (based on the frequency of wet containers in the shade vs. sun) indicating that *Ae. albopictus* did not specifically avoid containers in sunny areas (Wald χ^2^= 0.7; df=2; *P* = 0.68). The estimated organic content in the container was also not a predictor of the occurrence of *Ae. albopictus* (Wald χ^2^= 0.1; df=2; *P* = 0.94). The majority of the *Ae. albopictus*-positive containers surveyed (84.8%) were considered non-disposable, 11.4% were disposable, and 1 was a tree hole (0.4%) (Figure 2). Among the containers considered disposable, 10 of 12 snack bags (e.g. 453 g potato chip bags) sampled in August contained larvae.

Immature *Cx. pipiens* were significantly associated with several environmental factors (χ^2^= 19.9; df=3; P < 0.05). In contrast to *Ae. albopictus, Cx. pipiens* were positively associated with larger water volumes (large , χ^2^= 4.69, df=3, *P* < 0.05; very large , χ^2^= 4.75, df=3, *P* < 0.05). We also found a significant positive association between the presence of *Cx. pipiens* and exposed containers (sun, χ^2^= 7.83, df=2, *P* < 0.05). *Culex pipiens* displayed a non-random distribution across the different types of wet containers sampled (Table 4), with a higher than expected presence in buckets (χ^2^ =139.1; df =12; *P* <0.05), catch basins (χ^2^ =528.6; df =12; *P* <0.05), and pools (χ^2^ =160.8 ; df =12 ; *P* <0.05). 

Regression analysis performed on all *Ae*. *albopictus-*positive containers within the 50 meter buffer zone where BGS traps were located and the number of *Ae*. *albopictus* adults resulted in a lack of significant associations between larval indices and adult abundance (r = 0.14, r = 0.32, r =0.37 for HI, BI and CI, respectively, all P>0.05, Table 5). In contrast, when regression analyses were based only on positive *Ae*. *albopictus* key containers, we found significant relationships between larval based indices and *Ae*. *albopictus* adult numbers (r = 0.74, r = 0.74, r =0.72 for HI, BI and CI, respectively, all P<0.01, Table 5). Surprisingly, considering such a small percentage of positive containers, the nuisance threshold value of five adults on average across the 12 BGS traps in the site was surpassed six times between the end of July and mid-September [35]. The threshold value of five adults (male+female) was chosen based on the fact that 3 bites have been reported as a common nuisance threshold driving residents indoors (for more details please refer to [35]).

**Table 5:** Container and Breteau index profile for *Ae. albopictus* for each BGS trapping location using a 50 m radius buffer area during August 2009.

Trapping location	No. of containers with *Ae. albopictus*/(KEY)	^a^HI/^*^HI	^b^CI/^*^CI	^c^BI/^*^BI	Mean # of adult *Ae. albopictus*
BGS-1	3(1)	6.4/2.1	3/1.0	6.3/2.2	4.3
BGS-2	0(0)	0.0/0.0	0.0/0.0	0.0/0.0	5.5
BGS-3	7(3)	16.6/6.7	18.9/8.1	23.3/10.0	7.3
BGS-4	4(2)	6.5/6.5	5.19/2.6	12.9/6.5	10.0
BGS-5	6(5)	12.5/12.5	17.1/14.3	18.8/15.6	24.0
BGS-6	11(4)	18.6/6.9	15.9/5.8	25.5/9.1	7.0
BGS-7	6(2)	16.6/6.6	8.9/3.0	20/6.6	5.0
BGS-8	1(1)	2.6/2.6	0.5/0.5	2.6/2.6	2.3
BGS-9	3(1)	7.2/3.6	6.1/2.0	10.7/3.6	4.3
BGS-10	5(2)	22.7/9.1	6.1/2.4	22.7/9.1	7.0
BGS-11	0(0)	0/0.0	0.0/0.0	0.0/0.0	7.0
BGS-12	3(3)	10.0/10.0	3.2/3.2	10.0/10.0	18.3

Values limited to key containers (KEY) are also shown.

* Calculations based on key containers only.

a HI: the percentage of houses that are positive for larvae.

b CI: the percentage of water-holding containers that are positive for larvae.

c BI: the number of positive containers per 100 houses.

**Figure 4:**
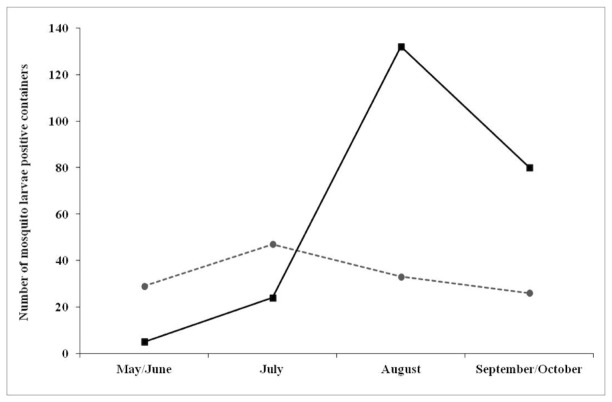
Seasonal shift in the number of containers positive for *Ae*. *albopictus* (black line) and *Cx. pipiens* (grey dashed line) during the larval surveys. Time stage 1 = May/June, 2 = July, 3 = August, 4 = September/October.

The distribution of larvae displayed seasonal trends, with species more concentrated in larger containers early in the season (64.8% of positive containers). August was the peak month for both *Ae. albopictus*-positive containers (132 out of 183, χ^2^ = 22.7; df =3; P <0.01) and *Ae. albopictus*-positive parcels (111 out of 825), while we collected the highest number of *Cx. pipiens* larvae in July (χ^2^ = 25.6; df =3; P <0.01) and detected a clear seasonal shift between *Ae. albopictus* and *Cx. pipiens* (Figure 4). Across the entire season, *Cx. pipiens* co-occurred with *Ae*. *albopictus* in only 3.7% of the positive containers (Table 3). 

## Discussion

Our study provides an account of the types of containers that are refractory to treatment in a representative portion of a typical inner-city urban neighborhood in the northeastern USA. Eventhough intensive and extensive source-reduction practices were being implemented monthly at the site, immature *Ae. albopictus* consistently occurred in medium volumes of water within buckets, tires, plant saucers, and in pockets of water in large pieces of equipment. The importance of these containers as sources of *Ae. albopictus* is supported by prior studies by Carrieri et al. [24] and Bartlett-Healy et al. [7], but our results demonstrate such containers are also particularly difficult to control. A characteristic common with many of these containers was the fact that they were in use by the homeowners (most of the positive buckets were recycling or trash containers) and therefore were not “trash”, a word often associated with sources of urban mosquitoes in public campaigns [47]. The robustness of our approach is underscored by our conclusion that *Cx. pipiens* instead thrives in larger volumes of water in pools and catch basins, a finding well established by others prior to us [24]. Somewhat contrary to expectations based on the aforementioned surveys by others, trash items such as snack bags, cans, and bottles were very rarely infested. Indeed, even though *Ae*. *albopictus* was the most common mosquito species associated with small trash, we had to inspect a very large number to find a few positive and all those were detected during the month of August when the abundance of *Ae. albopictus* was at its peak. Although there might be some value in inspecting small trash type containers during peak season in order to address problem areas, our findings suggest they do not constitute critical sources for this species. We hypothesize that the small volumes of water associated with these small items simply cannot sustain the development of *Ae. albopictus* from egg to adult even during a wet New Jersey summer [48], and are mostly ignored by ovipositing females except when pressure to avoid intraspecific competition forces them to seek sub-optimal sites (skip oviposition, Fonseca et al, unpublished results).

Our finding that immature *Ae. albopictus* were distributed randomly across shaded and exposed containers and among different levels of organic content appears to disagree with other studies that have reported preference for shaded containers [7,49] or those with medium to high organic content [27]. These different results may stem from our definition of “preference” that takes into consideration the frequency of each container type in the environment. In our survey, wet containers were significantly more common in the shade than in the sun, possibly because of lower evaporation rates [48]. Therefore, although *Ae. albopictus* were more often collected in the shade it was at a rate that matched container occurrence and therefore did not indicate preference. An alternative explanation for our findings is that the negative effect of UV light on the persistence of insecticides [50,51] results in higher survivorship in containers exposed to the sun, which would shift the occurrence of immature *Ae. albopictus* to sunny sites and mask female preference for containers in the shade. Nonetheless, it is clear that containers in the sun cannot be disregarded.

Additionally, although we systematically removed tires from the site each month [35], our finding that *Ae. albopictus* colonize tires at the frequency at which they occur in the environment indicates a rapid dynamic of tires in urban environments and aggressive use by *Ae. albopictus* [52]. In contrast, we found that *Ae. albopictus* were nearly absent from urban catch basins and rain gutters. This contrasts with findings that this species exploits catch basins in suburban Italy and Japan [53,54] and although that may reflect the bi-monthly treatment of catch basins with larvicides, we still found a significant presence of *Cx. pipiens* in catch basins but not *Ae. albopictus*. In contrast, the absence of *Ae. albopictus* from rain gutters agree with Obenauer et al. [49] and with the experimental predictions of Amerasinghe and Alagoda [55] that *Ae*. *albopictus* oviposits more often at ground level (<1 m) than at 3.5 and 7.0 m, the latter category matching the minimum height of rain-gutters in our study site, composed mostly of row homes with covered porches. These results indicate that *Ae. albopictus* may display consistent behavioral patterns (medium volumes of water, containers close to the ground), but also that the species may behave differently in urban and suburban settings, a line of research we are also pursuing actively (Fonseca et al. unpublished results).

The distribution of *Ae*. *albopictus* was highly seasonal, occurring in a relatively low number of containers from early May to July and increasing its presence in containers in August and September. In contrast, *Cx. pipiens* larvae were only common in the first half of the season (May to July). Our results are consistent with the findings of Carrieri et al. [24] and Costanzo et al. [26], who also reported a seasonal shift between these two important container species. Further investigation of the potential competition between *Ae*. *albopictus* and *Cx. pipiens* may elucidate patterns of West Nile virus transmission in the urban northeast since the expansion of *Ae. albopictus.*

Following our monthly intervention sampling of >20,000 wet containers during the five monthly surveys, revealed only 2.8% that were positive for mosquito larvae with *Ae. albopictus* present in 1.8% of containers, being by far the most abundant of eight species collected between May and October, 2009. However, even such a small percentage of positive containers was enough to generate nuisance levels (above our predetermined threshold of 5) of *Ae. albopictus*. This result underscores the conclusion that source-reduction and treatments implemented by professionals even at the aggressive rate only possible in the context of a federally funded program are not enough for area-wide control of *Ae. albopictus*. In addition, we found that most key container types for *Ae. albopictus* (buckets, plant saucers, and various types of equipment) are not abandoned or trash but are often actively used for a household purpose. These results increase the importance of mosquito awareness by residents of the key containers for *Ae. albopictus* production and the need for their cooperation to reduce mosquito populations. Residents can prevent larval development through such actions as weekly emptying of water, drilling holes for drainage, storage of equipment in a shed or garage, etc.

Supporting the impact of a small group of container types, we found that although basic larval-based indices were not correlated to local adult abundance, this result shifts dramatically to a significant correlation when only key positive containers are used for calculation of indices. This indicates that a few container types are critical sources of local adult populations of *Ae. albopictus*, and that focusing on them should be the most cost-effective strategy. However, in the past, targeted source reduction efforts have yielded only mixed outcomes [56,57]. In Brazil, after covering water tanks (holding 70% of *Ae. aegypti* pupae), researchers observed a significant and dramatic decrease in weekly adult *Ae. aegypti* collection, but in Thailand a targeted key container control campaign only achieved 15% reduction in the *Ae. aegypti* pupae per Person Index. Clearly further research is needed to assess if a targeted approach will be effective in reducing adult populations of *Ae. albopictus*, especially considering the observed flexibility this species exhibits with container choice. 

*Aedes albopictus* has become a widespread nuisance throughout the City of Trenton and across the highly urbanized Middle Atlantic States and its presence has been marked by an increase in residential public complaints [22]. We conclude that in urban areas, mosquito abatement personnel can ignore small trash items because *Ae. albopictus* seldom infests them and they are quickly replaced by residents at a rate that overwhelms the limitations of professional source-reduction campaigns [39]. Likewise both rain gutters and catch basins are not critical sources of *Ae. albopictus* in this area. Instead, in temperate North America, we suggest concentrating efforts in urban habitats with removal and management of buckets, plant saucers, tires, and equipment with medium sized pockets of water. This focus will also help in providing homeowners with a feasible action-plan since it is clear from our results that their continued intervention is required. We propose that targeting a small subset of containers for removal, and treatment in combination with multiple control measures such as ultra-low volume adulticiding, larviciding, and public education will lead to effective, economic, and sustainable integrated management of *Ae. albopictus* [35,33,37,39,58].
